# Integration of mobile sensors in a telemedicine hospital system: remote-monitoring in COVID-19 patients

**DOI:** 10.1007/s10389-021-01655-2

**Published:** 2021-10-15

**Authors:** Alexander Müller, Hannah Haneke, Valerie Kirchberger, Giulio Mastella, Michael Dommasch, Uta Merle, Oliver Heinze, Adonia Siegmann, Christoph Spinner, Alessandra Buiatti, Karl-Ludwig Laugwitz, Georg Schmidt, Eimo Martens

**Affiliations:** 1 Clinic for Cardiology, Klinikum rechts der Isar, Technical University Munich, Ismaninger Str. 22, 81675 Munich, Germany; 2 Board of Healthcare Management, Department Value-Based Healthcare, Charité - University Medicine, Berlin, Germany; 3 Department of Information Technology, Klinikum rechts der Isar, Technical University Munich, Munich, Germany; 4 Department of Gastroenterology and Infectious Diseases, University Hospital Heidelberg, Heidelberg, Germany; 5 Department Medical Information Systems, University Hospital Heidelberg, Heidelberg, Germany; 6 Clinic for Gastroenterology, Klinikum rechts der Isar, Technical University Munich, Munich, Germany; 7 German Center of Cardio-Vascular-Research (DZHK), Berlin, Germany

**Keywords:** Remote-monitoring, COVID-19, Telemedicine, Mobile sensors, Wearables, Digital health, Interoperability, Pandemic management

## Abstract

**Aim:**

The goal is to design and, in a next step, establish a scalable, multi-center telemonitoring platform based on existing systems for monitoring COVID-19 patients in home quarantine. In particular, the focus will be on raw data acquisition, integration of sensor data into the hospital system, structured data storage, and interoperability.

**Subject and methods:**

Data necessary for monitoring, otherwise provided in various portals, will be continuously queried and integrated into the hospital system via a new interface in this proof-of-concept work.

**Results:**

Based on extensive preliminary work at Klinikum rechts der Isar with a structured clinical database, we extend our system’s integration of raw data and visualization in dashboards, as well as scientific provision of data from mobile sensors for monitoring patients in home quarantine.

**Conclusion:**

Based on existing integrated telemonitoring systems supporting semantic and syntactic interoperability, short-term provision of scientific databases is possible. The integration of different mobile sensors into a clinical system for remote monitoring of patients around the clock is still new and to our knowledge unique.

## Introduction

The COVID-19 pandemic poses unprecedented and complex challenges to all healthcare systems that can only be addressed with innovative care delivery and integrated approaches. Several studies have demonstrated the feasibility and effectiveness of digital tools to address the multi-faceted challenges (Khorshid et al. 2020; Merle et al. 2020; Tayal et al. 2020) and the importance of integrating these solutions to realize their full potential.

Medical hardware and software solutions are a rapidly growing market worldwide; there are more than 362 different mobile health monitoring devices from 193 device families on the market (Muzny et al. 2020). In particular, the number and variety of mobile digital applications for remote monitoring has increased significantly. Remote monitoring—as part of telemedicine—is defined as the ambulatory monitoring of vital signs or device data via a telemedicine system without the patient being present in the clinic or practice. It should be emphasized that it is close-meshed monitoring with transmissions at least daily or, in the latest generations, every minute to continuous monitoring (Majumder et al. 2017). Real-time measurements are defined differently in medicine; in ultrasound diagnostics, for example, image processing in milliseconds is required (Von Ramm and Smith 1990). Telemedicine approaches have different technical realities such as battery life, connection quality, and others that determine the frequency of signal transmission. In high-security areas (e.g., patient monitoring), transmission times of up to 30 s should be maintained; in other areas, a few minutes is the current state of the art (Watson et al. 2020; Tayal et al. 2020).

While most remote monitoring solutions offer medically relevant information, an aggregation of all relevant medical information of a single patient is missing in most cases (Mishra et al. 2020). For mobile sensor data, each manufacturer provides a dedicated portal for visualization of sensor data, usually as a website. In our telemedicine center, patients with heart failure or implantable cardiac devices, among others, are monitored in addition to the data from the ear sensors. In everyday life, a large number of portals thus have to be monitored simultaneously. Here, there remains a need for further development of medical information systems.

The importance of managing the ever-increasing amount of digital information about a person’s health is particularly evident in situations that challenge even sophisticated healthcare systems, such as the COVID-19 pandemic. In the context of the pandemic, the focus was on developing solutions for early detection and efficient monitoring of patients with COVID-19 infection while creating systems that are open to integrating data from patients with other diseases (Hippchen et al. 2020; Smith et al. 2020). Effective use of telemedicine requires its appropriate integration as a “business as usual” modality in our healthcare system, the readiness of clinicians, and the acceptance of health care and healthcare systems organization (Harst et al. 2019; Hollander and Carr 2020; Smith et al. 2020). Finally, such a system must store data in a structured manner in a database and meet syntactic and semantic interoperability standards in order to use these data for rapid clinical and scientific evaluations (Henkel and Spinner 2020; Munos et al. 2016).

## Preliminary work

**Fig. 1:**
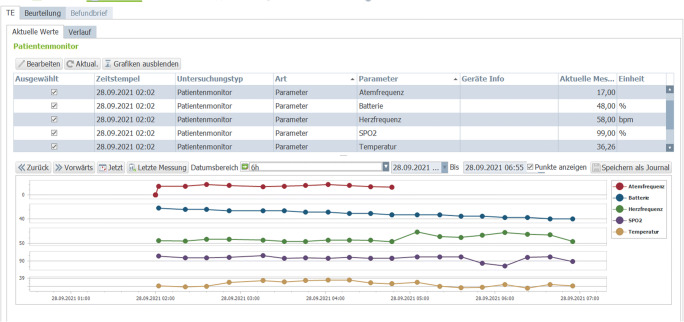
Integration of ear-sensor data into the CVIS

As part of a project funded by the German Research Foundation (DFG), a new cardiovascular information system (CVIS) with deep integration into the hospital information system (HIS) landscape was built at Klinikum rechts der Isar in Munich between 2017 and 2020. The focus was on the integration of different clinical medical devices. Because these devices mostly work with proprietary interfaces, the goal was to connect more than 60 different medical devices from different areas to a structured database system within three years and to fully automate the data exchange between the systems. The platform is based on a database that contains structured parameters, measured values and all raw biosignal data, thus enabling maximum semantic and syntactic interoperability in an open format. If already available, all stored parameters are mapped with corresponding LOINC CODES to make them available interoperable in the long term (Fig. 1).

## Objective

The aim of this project was to integrate data from mobile sensors for real-time monitoring into a clinic system, also for remote monitoring, and to show that continuous data transmission for patient monitoring is possible. The focus here is on providing users with the data in the familiar environment in the patient context without having to use different platforms in parallel. This results in the overarching objective of holistic patient care.

## Methods and implementation

**Fig. 2:**
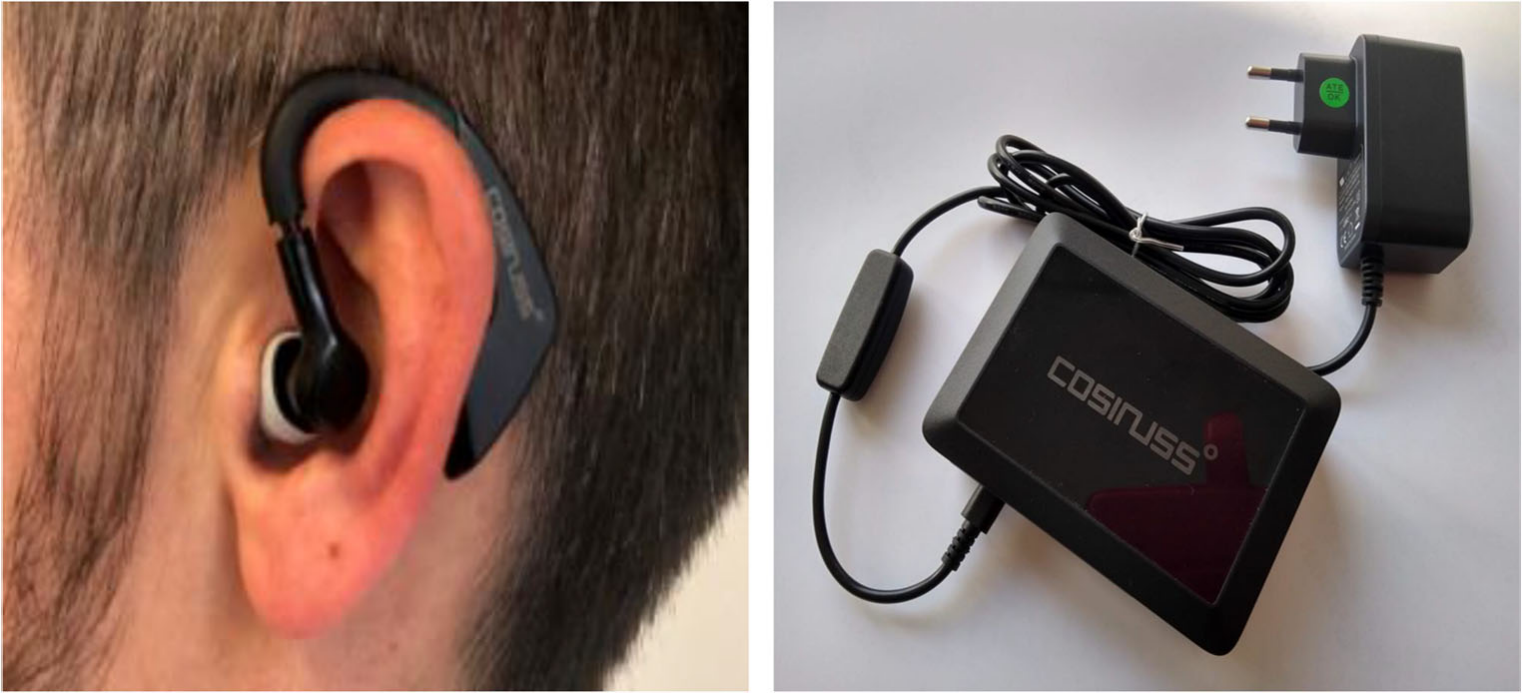
Left: ear-sensor; right: remote transmitter

For monitoring COVID-19 patients in home quarantine, we used the Cosinuss ear sensor because it can capture multiple measurements in one sensor (Cosinuss One, Fig. 2). The Cosinuss sensor can continuously monitor temperature, oxygen saturation, heart rate, and respiratory rate using photoplethysmography. These data are sent via Bluetooth to a transmitter or cell phone, from where they are encrypted and transmitted via the mobile network to a Cosinuss server. This data was monitored 24/7 at our telemedicine center. This facilitates detection of deterioration in the health status of individuals in home isolation and quarantine and enables rapid action, such as hospitalization, using previously defined standard operations procedures (SOPs).

To integrate the data into the hospital system, a service was programmed to retrieve data from all sensors every 60 s as a web service via a designated application programming interface (API); this time interval was chosen based on the limited existing work on the subject. For the device we used, data is transferred from the sensor to the platform every 15 min; therefore, for real time display, a minute-by-minute update is comparable to other patient monitoring systems with high safety requirements (Watson et al. 2020; Tayal et al. 2020).

To comply with data protection requirements—in particular the Bavarian Hospital Act—a procedure was developed in which all data on the manufacturer platforms can only be identified via the device serial number and thus no patient reference can be established; only in the hospital system is this data then assigned to the patient. In Bavaria, no patient data is allowed to leave the hospital (Tinnefeld et al. 2019). When the sensor is issued, the serial number of the sensor must be manually assigned to the patient once so that the server service then automatically assigns the collected data to the patient as a single examination time in a telemedicine module and visualizes it for the physician in a list of newly received examinations and in a dashboard. The assignment is done by scanning the bar code on the device’s packaging using a bar code scanner. In addition to the readings, the raw data signal (photoplethysmogram, PPG) is also transmitted and can be analyzed to assess signal quality if needed.

The focus of the integration was to store the data in a structured and interoperable way in the existing database so that it is also available for further analysis and is always visualized to the user in the same place in a normalized form. All imported values were mapped with corresponding existing fields in the database and associated LOINC codes.

In this proof-of-concept project, we were concerned with verifying a smooth transfer of the measured values via the API into the clinic system. All patients who were monitored by the telecenter using the ear sensor were included, after appropriate education. No patients were excluded from this technical study. The number of cases was previously set at 100 patients in order to produce a relevant amount of transmissions in case of missing comparative data. Using a predefined protocol, all transmissions in the manufacturer’s portal and in the clinic IT system were independently evaluated by a cardiologist and IT engineer and recorded accordingly. The parameters used were the completeness of the measurements and the time of the measurement. For each measurement, the time of import (timestamp) and the time of measurement were determined and compared. In addition, each measurement was checked for completeness of the transmitted parameters and raw data. Finally, the data were analyzed using R.

## Results

In this project, we were able to monitor 24/7 on 100 patients in the first and second waves of the COVID-19 pandemic in Germany. In total, the monitoring period for the included patients was seven months and three days. The average duration of monitoring per patient was 10.2 days. During this time, a total of 773,252 measurements were performed and transmitted (average 3621/day or 7732/patient). During each transmission, we were able to obtain all data transmitted from the sensor to the platform on the following parameters: battery capacity, signal quality index, oxygen saturation, heart rate, temperature, and respiratory rate (1 Hz sampling each, 225.6 MB) received without any problems. A total of 2614 h of raw data (3 x PPG and 1x time) were transmitted at 200 Hz sampling (15.06 GB in total).

In all (100%) cases, we were able to receive the transmissions of parameters and raw data within 1 min (58 s ± 18) after the measurement in our clinic system, and 100% of the transmitted data were correctly mapped by the interface into interoperable database fields (LOINC in this case) and visualized to the treating telemedicine physicians. The interface was thus transferred to our telemedicine platform in the hospital system and is currently being used to manage the pandemic in Bavaria.

In this project, we were able to show that it is technically possible to integrate data from mobile sensors with proprietary data formats into a clinic system and thus simplify real-time monitoring of COVID-19 patients in home quarantine.

## Discussion

Telemedicine has received a particular boost from the COVID-19 pandemic. From our point of view, it is important to test the functionality of these many new concepts, apps and sensors and to generate evidence for telemedicine applications. However, the basis for such studies must be structured and interoperable data collection, which we were able to demonstrate in this proof-of-concept project. Not only for scientific work, but also for a smooth and traceable telemedical treatment, an integration of the data into a clinical system certified as a medical device is necessary. One of the technical and acceptance barriers for the end user is the connection of the various mobile sensors to the clinical patient record. Our innovative project eliminates the highly inefficient dilemma of clinicians having to collect information from multiple, mostly web-based applications and often being forced to print it out to combine with the rest of the clinical information in a paper patient record, resulting in poorer patient outcomes and higher costs. In our view, meaningful treatment for physicians is only possible with an integration that allows physicians to view the various mobile data in a structured way, e.g., from the sensor technology described above, device therapy, or mobile heart failure monitoring with body scales and blood pressure measurement (Harst et al. 2019; van Dyk 2014).

In connecting mobile sensors, we faced the problem of lack of standardization and implementation of proprietary interfaces to the respective platforms or devices. In the long term, this can only be done in collaboration with the manufacturers. Only in a few cases, it is possible to benefit from standardized data extraction (e.g. FIHR). We were able to solve this problem by programming this integration and make the proprietary data available in an interoperable way.

To our knowledge, there is currently no telemedicine unit in Germany where it is possible to integrate raw data and measured values from different telemedicine sensors into the clinic system, process them in a normalized way for the user, and make them available for structured reporting or treatment. With this integration, we are closing an important gap in telemedical care while complying with all current data protection regulations. The connection can be used as a template for a wide range of mobile sensors.

## Summary

Our goal in the pandemic was to expand a remote monitoring unit based on extensive preliminary work on integrating mobile sensor data. In the context of the first wave of the COVID-19 pandemic, we were able to demonstrate that we can make a digital contribution to the management of the pandemic in the short term. The focus of this work is on raw data collection, structured data storage, and interoperability. Based on extensive preliminary work at Klinikum rechts der Isar with a structured clinical database, a biosignal repository and the pseudonymized science server, we were able to integrate an ear sensor for monitoring COVID-19 patients into our system in the short term—in addition to the already integrated mobile sensors—and make it available for continuous and technically secure monitoring in the telemedical center.

The integration of such a continuous ambulatory monitoring into a hospital system is so far new and to our knowledge unique in Germany. This system will be extended to other hospitals and practices to cope with the pandemic and may later also contribute to better treatment of other chronic diseases.
